# Charting a course for smartphones and wearables to transform population health research

**DOI:** 10.2196/preprints.42449

**Published:** 2023-02-07

**Authors:** William G Dixon, Sabine N van der Veer, Syed Mustafa Ali, Lynn Laidlaw, Richard JB Dobson, Cathie Sudlow, Tim Chico, Jacqueline AL MacArthur, Aiden Doherty

**Affiliations:** 1 Centre for Epidemiology Versus Arthritis, Manchester Academic Health Science Centre, The University of Manchester, Manchester, UK; 2 Centre for Health Informatics, Manchester Academic Health Science Centre, The University of Manchester, Manchester, UK; 3 Public contributor; 4 Department of Biostatistics and Health Informatics, Institute of Psychiatry, Psychology and Neuroscience, King’s College London, London, UK; 5 British Heart Foundation Data Science Centre, Health Data Research UK, London, UK; 6 Big Data Institute, Nuffield Department of Population Health, University of Oxford, Oxford, UK

## Abstract

The use of data from smartphones and wearable devices has huge potential for population health research given high device ownership, the range of novel health-relevant data types available from consumer devices, and the frequency and duration over which data are, or could be, collected. Yet the uptake and success of large-scale mobile health research in the last decade has not matched the hyped opportunity. We make the argument that digital person-generated health data is required and necessary to answer many top priority research questions through illustrative examples taken from the James Lind Alliance Priority Setting Partnership. We then summarise the findings from two UK initiatives that considered the challenges and possible solutions for what needs to be done, and in what way, to realise the future opportunities of digital person-generated health data for clinically important population health research. Examples of important areas to be addressed to advance the field include digital inequality and addressing possible selection bias, easy access for researchers to the appropriate data collection tools including how best to harmonise data items, analysis methodology for time series data, methods for patient and public involvement and engagement to optimise recruitment, retention and public trust, and providing greater control of their data to research participants. There is also a major opportunity through the linkage of digital persongenerated health data to routinely-collected data to support novel population health research, bringing together clinician-reported and patient-reported measures. We recognise that well conducted studies need a wide range of diverse challenges to be skilfully addressed in unison: for example, epidemiology, data science and biostatistics, psychometrics, behavioural and social science, software engineering, user interface design, information governance, data management and patient and public involvement and engagement. Consequently, progress would be accelerated by the establishment of a new interdisciplinary community where all relevant and necessary skills are brought together to allow excellence throughout the lifecycle of a research study. This will require a partnership of diverse people, of methods and of technology. Get this right and the synergy has the potential to transform many millions of people’s lives for the better.

## Introduction

Consumer digital devices provide a major opportunity to transform our understanding of the mechanisms, determinants and consequences of diseases ranging from arthritis to dementia to heart disease [1][2][3]. Most people in developed and developing societies now own - and regularly use - consumer digital devices. Around nine in ten people own a smartphone in the UK [4], while a fifth of US adults own wearable technology like smartwatches and fitness trackers [5]. Device ownership means many people could contribute to health research from the comfort of their home, offering small amounts of time to share data to help address clinical questions that matter to them.

Considering the wide range of types of data available, and the frequency and duration over which they are or could be collected, a significant step change in how we conduct health research is within reach. Such data provides a much clearer picture of the daily rhythms of health, wellbeing and disease, as well as the environment in which these occur. The touchscreens, motion sensors, microphones, cameras, location sensors and other technologies within the devices allow us to rethink how we measure things that are important and relevant to health research. Consider the measurement of physical activity as an example. It is an important risk factor for many diseases, while also being negatively impacted when living with a condition such as arthritis or stroke. Wrist-worn devices offer an opportunity to shift from using subjective questionnaires asking people to report “*In a typical week, on how many days did you do 10 minutes or more of moderate physical activities like carrying light loads, cycling at normal pace?*” [6] to the continuous objective measurement of physical activity patterns [7]. One can easily see the difference in granularity, validity, reliability, and data collection burden between these two methods.

Smartphones and wearables have, however, not been used for research at scale beyond a handful of high-profile studies. Two of the better examples of large scale studies are the COVID Zoe study which demonstrated that mass collection of digital person-generated health data is both feasible and valuable, providing important early evidence for public health that anosmia was a key symptom of COVID [8]. The Apple Watch study proved smartwatches can detect clinically meaningful heart rhythm patterns like atrial fibrillation [9]. But despite these studies illustrating major potential for answering important research questions at speed and scale, this opportunity is yet to be fully exploited. Furthermore, no large-scale study has yet established linkage of longitudinal wearable data to major clinical outcomes. Such linkage is important as it brings together key ingredients for important population health research questions: for example, it would allow us to understand whether digital interventions to improve physical activity lead to improvements in hard clinical outcomes like a reduction in myocardial infarctions, or a reduction in the number of people who develop diabetes.

In this editorial, we make the case that there remains a critical need to collect and link digital person-generated health data at scale by illustrating that it is *required and necessary* to answer many vital research questions that matter to patients, clinicians and policy makers, and describe the requirements to deliver this. We then summarise what is needed to advance progress in this important and emerging field.

## Opportunities

To illustrate the importance and need for digital person-generated health data, we reviewed priority research questions from a number of common conditions. The James Lind Alliance is a UK initiative that brings together patients, carers, clinicians and researchers in Priority Setting Partnerships to identify and prioritise the top 10 most important unanswered questions or uncertainties for a given disease area [10]. While there are other means to identify research priorities, the James Lind Alliance follows a standardised process that is common across diseases, plus it brings together the views of different stakeholders. We reviewed the top 10 question lists for six common disorder areas: arthritis, diabetes, chronic obstructive pulmonary disease, inflammatory bowel disease, stroke, and mental health. Each disorder area contained at least one question (and often several) that would be optimally addressed with digital person-generated health data, with or without additional linked clinical data. Box 1 contains some of these questions, showing the need to collect physical and mental health symptoms, and environmental factors such as diet and exercise.

A recent review of what happens after a priority setting exercise [17] noted that addressing a priority topic requires researchers to design a dedicated study. The opportunity to collect data direct from patients at scale via digital devices could now help researchers and the public to address many of these top priority questions more easily and robustly. But before we can harness this potential, we need to chart a course to overcome the barriers to conducting such large-scale population health research well.

We ran two parallel and complementary initiatives in 2021 to investigate possible solutions to successfully use smartphones and wearable data in population health research. The first was a British Heart Foundation (BHF) Data Science Centre workshop which focussed on wearables for cardiovascular research [18]. The second was a roundtable event considering the future of digital person-generated health data for UK health research, hosted by the Centre for Epidemiology Versus Arthritis [19]. Both brought together multiple stakeholders including patients, healthcare professionals, researchers, funders, policy makers, governance experts, and industry representatives, reflecting the importance of widespread consultation. The two workshop reports underline the major opportunities for population health research using digital person-generated health data. They both also recognise that countries such as the UK are in a particularly strong position given the possibility of linking person-generated health data with routinely collected health data such as that from the National Health Service (NHS) with its universal access to healthcare and ‘cradle-to-grave’ health records. There is a pressing need for national-scale studies in which large numbers of smartphone and wearable users are invited to consent to share their device data to allow this to be linked to their routinely collected healthcare information for research. This mobile data could enhance population health research by integrating with national investments in digital infrastructure to support health data research [20], as well as in large population cohort studies with genetic and deep phenotypic information, such as UK Biobank [21] and Our Future Health [22].

## Requirements

Well conducted population health research must consider potential challenges during study design and how to navigate them – a key area of discussion in both workshops. Recruitment of study participants based on device ownership will be skewed as not everyone owns a device, introducing possible selection bias: for example, people who use wearable activity trackers are more active, younger, and more affluent than those who do not [23]. Study results must be useful and ideally generalisable to a wider population. It is vital that research does not worsen already existing health, social and racial inequalities [24]. Researchers need to be able to set up studies easily and efficiently, including high-quality study design and access to the right data collection tools that are both stable and flexible [25]. Data harmonisation and interoperability are important challenges: the proliferation of devices with different proprietary software algorithms to determine measures like step count creates a risk that researchers cannot trust the outputs of consumer devices. Different devices provide different step counts for the same activity and vary greatly in accuracy [26]. There is a need to generate reproducible digital phenotypes, from raw sensor data and low-level features (e.g. measures of mobility, or sleep), as well as understanding the environment and context in which data is generated which may need more qualitative approaches. There is also a need for harmonisation of self-reported information such as symptoms within and across diseases, especially as the number of people with multiple long-term conditions increases [27]. Public trust, engagement and involvement is essential from the earliest point. This includes defining and prioritising the most important, relevant and feasible questions to address, designing the most appropriate studies, co-designing userfriendly devices and apps [28], inviting people to join a study through the remote consent process [29], and keeping them motivated to optimise ongoing engagement [30]. It is also important to enable participants to maintain and feel in control of where and how their data is used, and share the benefits and results of their contributions [31].

## Proposed solutions

Realising the potential of patient-generated data in healthcare research requires a new interdisciplinary community to be established. Academics from diverse areas such as epidemiology, software development, data science and biostatistics, psychometrics, behavioural and social science need to work with patients and healthcare professionals, alongside colleagues from industry who could contribute skills such as hardware and software engineering, user interface design, cyber-security and data management. Only by operating across disciplinary boundaries can we develop the foundations for future high-quality research, and in turn support a wider group of interested - but so far relatively inexperienced – researchers. This can be done by defining and supporting best practice, and providing access to the tools and methods needed to address the highest priority questions.

In countries such as the UK, a crucial requirement is to understand how we can best link digital person-generated health data with national healthcare datasets for research, in a way that is understandable, feasible and acceptable to participants, and allows them the option of retaining control of how and by whom their data is used. This linkage should use existing national infrastructure, such as trustworthy research environments [32]. In addition to the technical infrastructure, it also requires the development and evaluation of a range of approaches and methods. For example, how best to recruit and remotely consent participants, securely store and link the different data types across different geographical areas, ensure the validity and harmonisation of data across devices, and engage participants through feedback and providing control to ensure we maintain trust. In this context, prominent involvement of patients and the public is the most vital factor as we proceed: we can only undertake large scale population health research if people are willing to participate, consent, collect and share their data, often repeatedly over time. Before asking this of patients and the public, we must ensure research is done in a way that is acceptable and valuable, and has meaning and relevance to them [33][34].

We believe the time is right to create the partnerships, platforms, tools and methods to allow us to collect data directly from patients via digital devices, securely link this to their routinely collected healthcare data in a trustworthy way, and answer many more questions that matter to patients, healthcare professionals, policymakers and the wider public.

## Box 1 Examples of as-yet unanswered questions that digital person-generated health data would optimally address as part of the solution.

Questions come from James Lind Alliance priority setting exercises for six common disorder areas.

1) “Is regular exercise and physical activity effective at reducing disease progression [in **hip and knee osteoarthritis**]?” [11]
2) How do stress and anxiety influence the management of **type 2 diabetes** and does a positive mental wellbeing have an effect?” [12]
3) “What is the best way to tell the start of an exacerbation [of **chronic obstructive pulmonary disease**] from day-to day variation in symptoms?” [13]
4) “What role does diet have in the management of mildly active or inactive **ulcerative colitis or Crohn’s Disease** to achieve normal daily activities and symptom control?” [14]
5) “How common are psychological problems and what impact do they have on the lives of people affected by **stroke**?” [15]
6) “How do certain mental health conditions (e.g. **depression**) affect how people engage with technology?” [16]
